# Tularemia on Martha’s Vineyard: Seroprevalence and Occupational Risk

**DOI:** 10.3201/eid0903.020462

**Published:** 2003-03

**Authors:** Katherine A. Feldman, Donna Stiles-Enos, Kathleen Julian, Bela T. Matyas, Sam R. Telford, May C. Chu, Lyle R. Petersen, Edward B. Hayes

**Affiliations:** * Centers for Disease Control and Prevention, Atlanta, Georgia, USA; † Martha’s Vineyard Hospital, Oak Bluffs, Massachusetts, USA; ‡ The Massachusetts Department of Public Health, Boston, Massachusetts, USA; § The Harvard School of Public Health, Cambridge, Massachusetts, USA

**Keywords:** tularemia, *Francisella tularensis*, seroepidemiologic studies, prevalence, antibodies, research

## Abstract

We conducted a serosurvey of landscapers to determine if they were at increased risk for exposure to *Francisella tularensis* and to determine risk factors for infection. In Martha’s Vineyard, Massachusetts, landscapers (n=132) were tested for anti–*F. tularensis* antibody and completed a questionnaire. For comparison, serum samples from three groups of nonlandscaper Martha’s Vineyard residents (n=103, 99, and 108) were tested. Twelve landscapers (9.1%) were seropositive, compared with one person total from the comparison groups (prevalence ratio 9.0; 95% confidence interval 1.2 to 68.1; p=0.02). Of landscapers who used a power blower, 15% were seropositive, compared to 2% who did not use a power blower (prevalence ratio 9.2; 95% confidence interval 1.2 to 69.0; p=0.02). Seropositive landscapers worked more hours per week mowing and weed-whacking and mowed more lawns per week than their seronegative counterparts. Health-care workers in tularemia-endemic areas should consider tularemia as a diagnosis for landscapers with a febrile illness.

Tularemia is a potentially severe zoonosis caused by *Francisella tularensis*, a small, pleomorphic, gram-negative bacterium. The bacterium can be transmitted by an arthropod bite, ingestion, inhalation, or direct contact with infected tissues. The clinical signs and symptoms of tularemia depend, in part, on the route of injection. The ulceroglandular form, in which an ulcer develops at the site of inoculation and is accompanied by regional lymphadenopathy, is the most common. The less common but more severe primary pneumonic form develops after inhalation of the bacteria; pneumonic tularemia can be difficult to diagnose because the respiratory signs and symptoms may be minimal or absent and, when present, are often nonspecific. The typhoidal form of tularemia has no localizing signs and is, therefore, also often difficult to diagnose. Tularemia can also occur in glandular, oculoglandular, and oropharyngeal forms. An average of 124 cases of tularemia was reported annually in the United States from 1990 to 2000 (*1*).

Tularemia is endemic on Martha’s Vineyard, an island off the coast of Cape Cod, Massachusetts. The only two reported outbreaks of pneumonic tularemia in the United States occurred on Martha’s Vineyard in 1978 and 2000 (*2*,*3*). During the outbreak in the summer of 2000, 15 patients with tularemia were identified; 11 had pneumonic tularemia. A case-control study demonstrated that lawn mowing or brush-cutting were risk factors for pneumonic tularemia (adjusted odds ratio 6.7; 95% confidence interval [CI] 1.1 to 39.9). Five patients were professional landscapers, and patients with pneumonic tularemia were approximately 32 times more likely to have worked as a landscaper than controls were (*3*).

Tularemia transmission on Martha’s Vineyard continued in the summer of 2001; one case of ulceroglandular and three cases of primary pneumonic tularemia were identified. Of the patients with pneumonic tularemia, two were professional landscapers, and one was a farmer who had mowed fields 4–6 hours a day the week before illness. We conducted a serosurvey to determine the prevalence of antibodies to *F. tularensis* among landscapers and three comparison groups and to evaluate potential risk factors for exposure to *F. tularensis* among landscapers.

## Methods

In July 2001, landscapers on Martha’s Vineyard were offered free testing for anti-*F. tularensis* antibody during an all-day event publicized at a local small engine-repair shop and through community-wide advertisements. After providing informed consent, participating landscapers gave serum samples and completed a risk factor questionnaire about their professional activities, contact with animals and arthropods, and past medical history. A professional landscaper was defined as anyone who reported their occupation as landscaper, tree worker, property manager, caretaker, professional gardener, or land or lot clearer.

For comparison, serum samples were obtained from three control groups. Group 1 (n=103) comprised nonlandscaper patients at two local physicians’ offices who were having blood drawn for other reasons (n=56), nonlandscaper members of various Martha’s Vineyard civic organizations (n=27), and persons who participated in our serosurvey but did not meet the definition of landscaper (n=20); all participants gave informed consent. Groups 2 and 3 comprised individual serum samples from anonymous, healthy Martha’s Vineyard residents who had blood drawn for other reasons (n=99 in July and n=108 in October). All serum samples were tested at the Centers for Disease Control and Prevention (CDC) for anti–*F. tularensis* antibodies with a microagglutination assay (*4*); titers of at least 1:128 were considered positive.

The seroprevalence of antibody to *F. tularensis* in landscapers was compared to the seroprevalence in each of the three control groups. Seropositive landscapers were compared to seronegative landscapers to determine risk factors for seropositivity. Statistical analyses were performed in Epi Info 2000 (CDC, Atlanta, Georgia) and SAS version 8 (SAS Institute, Inc., Cary, NC). For univariate analyses, prevalence ratios were determined for dichotomous variables; the Mann-Whitney U test was performed to compare the median values of the continuous variables. Multivariable logistic regression was used to determine significant associations with seropositivity while controlling for variables that were significant on univariate analyses. Forward, backward, and stepwise selection procedures were used to obtain a parsimonious model with variables that were significant on univariate analysis.

A CDC ethics review coordinator reviewed the study plan and determined that the survey represented a public health response that did not require additional ethics review. The Harvard School of Public Health Institutional Review Board also approved our serosurvey as part of a broader study of zoonotic diseases in North Atlantic communities.

## Results

One hundred thirty-two landscapers requested serologic testing and completed risk factor questionnaires. These landscapers included 117 persons who described their occupation as landscaper on the questionnaire and 15 persons who listed their occupations as tree worker, property manager, caretaker, professional gardeners, or land or lot clearer. Compared to persons in the control groups, the landscapers were younger (median 37 years of age, compared with median 58, 49, and 50 years for groups 1, 2, and 3, respectively) and more likely to be male (79%, compared with 60%, 49%, and 41%, respectively, in the control groups). Of the 56 persons in control group 1 who were enrolled at local physicians’ offices, 27 (48%) went to their physician for a complete physical exam, 23 (41%) had an office visit, and 6 had no recorded reason for the office visit. None of these 56 patients reported a febrile illness.

Twelve (9.1%) of the 132 landscapers were seropositive for anti–*F. tularensis* antibodies (titer range 1:256–1:2048), compared to one person total from the three control groups (titer 1:128). The seropositive control sample was from a healthy Martha’s Vineyard resident who had blood drawn in July (group 2). Compared to control group 2 (99 residents who had blood drawn in July), Martha’s Vineyard landscapers were nine times more likely to be seropositive (95% CI 1.2 to 68.1; p=0.02) (Table 1). All 12 seropositive landscapers described their occupation as landscaper on the questionnaire, and they reported working as landscapers for 2–52 years (median 11 years). Two seropositive landscapers reported having been diagnosed with tularemia by a physician (one in 1985 and one in 1986); two others reported having had an undiagnosed febrile illness in 2000 or 2001. (We did not ask about febrile illnesses before 2000 because of concern about recall bias.)

**Table 1:** Relative seropositivity of Martha’s Vineyard landscapers compared with three control groups, Martha’s Vineyard, 2001^a^

Population	Seropositive landscapers/total no. (%)	Seropositive controls/total no. (%)	Seroprevalence ratio (95% CI)	Yates corrected p value
1) Landscapers vs. physicians’ office patients and members of civic organizations	12/132 (9.1)	0/103 (0)	Undef (undef, undef)	0.004
2) Landscapers vs. residents (July)		1/99 (1)	9.0 (1.2 to 68.1)	0.02
3) Landscapers vs. residents (October)		0/108 (0)	Undef (undef, undef)	0.004

^a^CI, confidence interval; undef, undefined.

Of the 12 seropositive landscapers, 11 were male; seropositive and seronegative groups had no substantial difference in the proportion of males. The median age of seropositive landscapers was 35 years of age (range 18–66 years), compared with a median of 38 years of age (range 12–75 years) in seronegative landscapers (p=0.83). Of landscapers who used a power blower, 15% (11/72) were seropositive, compared to 2% (1/60) of landscapers who did not use this tool (prevalence ratio 9.2; 95% CI 1.2 to 69.0; p=0.02) (Table 2). Of 132 landscapers, 116 (88%) mowed lawns, and 106 (80%) used a weed-whacker. Seropositive landscapers worked more hours per week mowing (median 29.5 vs. 15 hours; p=0.03) and weed-whacking (median 10 vs. 3 hours; p=0.01) and mowed more lawns per week (median 25 vs. 3 lawns; p=0.0003) than their seronegative counterparts (Table 3). Seropositive and seronegative landscapers reported similar frequencies of exposure to arthropods or sick or dead mammals (Table 2). A multivariable logistic regression model was constructed by using all variables significant on univariate analysis (power blower use, number of lawns mowed, hours mowed per week, and hours weed-whacked per week). No single variable was significantly associated with seropositivity after adjustment for the effects of all other variables. When forward, backward, and stepwise selection procedures with a 0.05 significance requirement for inclusion in the model were used, the final model contained only the number of lawns mowed per week (OR=1.04; 95% CI 1.01 to 1.07; p=0.004).

**Table 2:** Risk factors among landscapers (dichotomous variables), Martha’s Vineyard, 2001^a^

Potential risk factor	Seropositive among exposed no. (%)	Seropositive among unexposed no. (%)	Prevalence ratio (95% CI)	Yates corrected p value
Mow or brush-cut during summer	12/116 (10.3)	0/16 (0)	Undef (undef, undef)	0.38
Recall mowing or brush-cutting over dead animal	4/30 (13.3)	8/79 (10.1)	1.3 (0.4 to 4.1)	0.90
Use power blower during summer	11/72 (15.3)	1/60 (1.7)	9.2 (1.2 to 69.0)	0.02
Ticks crawling on body	10/112 (8.9)	2/18 (11.1)	0.8 (0.2 to 3.4)	0.89
Ticks attached to skin	4/73 (5.5)	8/59 (13.6)	0.4 (0.1 to 1.3)	0.19
Seen sick or dead rabbits in past year	6/60 (10)	6/71 (8.5)	1.2 (0.4 to 3.5)	0.99

^a^CI, confidence interval; undef = undefined.

**Table 3:** Risk factors among landscapers (continuous variables), Martha’s Vineyard, 2001

Exposure	n	Mean	Median	Mann-Whitney p value
Average hrs mowing/wk				
Seropositive	12	35	29.5	
Seronegative	118	21	15	0.03
Average hrs weed-whacking/wk				
Seropositive	11	26	10	
Seronegative	114	9	3	0.01
Average no. lawns mowed/wk				
Seropositive	11	31	25	
Seronegative	112	12	3	0.0003

Forty-one percent of landscapers reported that they wore a mask either sometimes or always while performing landscaping activities in 2001, compared with 23% in 2000 (p=0.005). However, few landscapers reported always wearing a mask in either year (3% in 2000 and 6% in 2001). Ninety-two percent of seropositive landscapers reported never wearing a mask in 2000, and 58% reported never wearing a mask in 2001; these proportions were not significantly different from seronegative landscapers. When mask-wearing was dichotomized into wearing a mask always versus sometimes or never, no significant differences between seropositive and seronegative landscapers occurred in either year.

## Discussion

In 2001, after 2 years of increased tularemia transmission on Martha’s Vineyard, 9.1% of 132 tested landscapers on the island were seropositive for anti–*F. tularensis* antibody, compared with <1% of nonlandscaper residents in each of three comparison groups. The seroprevalence observed in landscapers is comparable to that described in groups traditionally considered at risk for tularemia; for example, 2.4% to 17.5% of Native Americans and trappers in North America have been reported to have detectable antibody to *F. tularensis* (*5*–*12*). In Europe, where only the milder type B *F. tularensis* is found, seroprevalence estimates of 9.7% and 19.7% have been reported among populations affected by outbreaks (*13*). Estimates of tularemia seroprevalence in the general populations of North America and Sweden have been reported to range from 0% to 1.8% (*9*,*13*–*15*). While agglutination tests were also used in these earlier reports to determine antibody levels, different reagents and techniques may have been employed. In addition, the cutoff for a positive result was generally set much lower (often >1:8 or >1:20) than that used by CDC, with a potential loss in test specificity and exaggerated reported seroprevalence.

Historically, disproportionate numbers of tularemia cases have been reported among laboratory workers, farmers, veterinarians, sheep workers, hunters or trappers, and cooks or meat handlers (*16*). Our results indicate that landscapers on Martha’s Vineyard have increased exposure to *F. tularensis*. Of the eight sporadic case-patients identified in the tularemia outbreak on the island in 1978, two were gardeners (*2*). Sporadic tularemia cases in landscapers or persons participating in landscaping activities in Colorado and South Carolina suggest that this increased exposure is not unique to Martha’s Vineyard (*17*,*18*). Health-care workers in tularemia-endemic areas should consider a diagnosis of tularemia in landscapers who have fever or pneumonia.

Arguably, landscapers are more likely to be exposed to *F. tularensis* because they spend most of their time outdoors and are thus more likely to encounter infected ticks and animals. The case-control study conducted in the summer of 2000 showed an association between pneumonic tularemia and mowing or brush-cutting activities, but case-patients and controls did not differ in their exposure to ticks and animals (*3*). In 2001, seropositive landscapers were more likely to have used a power blower, spent more hours mowing and weed-whacking, and mowed more lawns than seronegative landscapers, but the groups did not differ in frequencies of exposure to arthropods or sick or dead mammals. Mowing or brush-cutting was not significantly associated with seropositivity when analyzed as a dichotomous variable, which may be caused by a lack of ability to detect a significant difference since only 16 of 132 landscapers did not mow lawns. The number of lawns mowed was the factor most robustly associated with seropositivity in our study, but the small number of seropositive landscapers limits the ability to detect other significant differences. The association between seropositivity and increased participation in potential aerosol-generating activities in the absence of an association with arthropod or animal exposure further supports the hypothesis that *F. tularensis* persists in the environment and is aerosolized and inhaled during mowing activities. Lawn mowing has previously been implicated in an outbreak of *Chlamydia psittaci* (*19*), and infection with *Legionella* spp. has been attributed to aerosolization of the organism in potting soil during gardening activities (*20*).

The clinical manifestations and severity of illness after infection with *F. tularensis* depend on the portal of entry, infectious dose, virulence of the organism, and immune status of the infected person; despite the organism’s high infectivity, asymptomatic infection with *F. tularensis* is known to occur. Eight of the seropositive landscapers did not report a previous diagnosis of tularemia or recall an undiagnosed febrile illness in 2000 or 2001. Because of concerns about recall bias, we did not ask about an undiagnosed febrile illness before 2000; thus, some of these eight landscapers might have had such an illness, which resolved without intervention or was treated empirically with an agent effective against many tick-borne infections. Some or all of them may also have had an asymptomatic infection. Many of the seropositive Native Americans and trappers previously surveyed in North America also did not recall clinical illness (*5*,*6*), yet at least some of them were likely infected with the more virulent type A *F. tularensis*. To date, only type A *F. tularensis* has been isolated from Martha’s Vineyard specimens (*2*,*3*), including one isolate from a patient who died of pneumonic tularemia in 2000, and one isolate recovered from a dead rabbit in 2001.

Efforts on Martha’s Vineyard to prevent tularemia should focus on landscapers who participate in aerosol-generating activities, as well as other persons who mow many lawns. Preventive efforts should include educating landscapers to survey their work areas for carcasses or excreta, and if encountered, to avoid or properly dispose of them. Equipment should be maintained in good working order; for example, the protective skirting and collection bags found on mowers should be kept intact. Landscapers not already using respiratory protection might consider doing so when generating aerosols. Following increased awareness of tularemia in 2000, landscapers did increase their use of respiratory protection; however, the effectiveness of masks in preventing occupational exposure to *F. tularensis* has not been evaluated. Since none of the seropositive landscapers reported always wearing a mask in either 2000 or 2001, we cannot draw any conclusions about the potential protective effect of masks from our data. Seronegative landscapers wore masks at the same low frequency, and exposure of the seropositive landscapers might have occurred in the past, before they became aware of the potential benefits of mask-wearing. Landscapers should be made aware of the risk for tularemia and should seek prompt medical attention if a febrile illness develops after they participate in aerosol-generating activities. We have shown that landscapers are at increased risk for infection with *F. tularensis*; however, some patients in the 2000 outbreak had mowed only their own lawns. The recommendations for landscapers apply to all persons who mow lawns.

Several possible limitations to this study exist. Both our landscaper and control populations were enrolled through convenience sampling and may or may not be representative of all landscapers on Martha’s Vineyard or the general population of Martha’s Vineyard residents. Samples from persons in control groups 2 and 3 were anonymous; therefore, we have no information on the occupations of those persons. Persons in these groups may be landscapers and could even be in our landscaper series. If any of those persons were landscapers, nondifferential misclassification occurred and would bias our results to the null, so the actual prevalence ratios could be stronger than what we observed. Landscapers on the island include both permanent and seasonal residents and determining the size of the total landscaper population is not possible; therefore, knowing what proportion of landscapers participated in our study is also not possible. The small number of seropositive landscapers limited statistical power for risk factor analyses and multivariable analysis, and our cross-sectional study design did not permit us to assess temporal relationships between exposure to potential risk factors and seropositivity.

Professional landscapers on Martha’s Vineyard are a newly identified occupational group with increased exposure to *F. tularensis*. Landscapers appear to be at least nine times more likely to have measurable anti–*F. tularensis* antibodies than nonlandscapers, and seropositive landscapers mow more lawns per week than seronegative landscapers. Health-care workers in tularemia-endemic areas need to be aware of this occupational risk when evaluating landscapers with a febrile illness. Landscapers in tularemia-endemic area should be aware of the potential risk of acquiring infection and should seek prompt medical attention if a febrile illness develops after landscaping activities.
